# Bictegravir concentrations in breastmilk of healthy, lactating women without HIV

**DOI:** 10.1093/jac/dkag022

**Published:** 2026-02-02

**Authors:** L C van der Wekken-Pas, E van Leeuwen, E W J van Ewijk-Beneken Kolmer, D M Burger, A C Colbers

**Affiliations:** Department of Pharmacy, Pharmacology and Toxicology, Radboudumc, Postbus 9101, Nijmegen 6500 HB, The Netherlands; Department of Obstetrics and Gynecology, Reproduction and Development, Amsterdam UMC, Amsterdam, The Netherlands; Department of Pharmacy, Pharmacology and Toxicology, Radboudumc, Postbus 9101, Nijmegen 6500 HB, The Netherlands; Department of Pharmacy, Pharmacology and Toxicology, Radboudumc, Postbus 9101, Nijmegen 6500 HB, The Netherlands; Department of Pharmacy, Pharmacology and Toxicology, Radboudumc, Postbus 9101, Nijmegen 6500 HB, The Netherlands

## Abstract

**Introduction:**

Risk of transmission of HIV through breastfeeding is minimal in case of maternal viral suppression due to antiretroviral therapy. However, to what extent antiretroviral drugs transfer into breastmilk is not fully understood. Data are especially lacking for relatively newer drugs—such as bictegravir—that only recently was approved to use in pregnancy. Therefore, the aim of this study is to determine infant exposure to bictegravir through breastmilk.

**Materials and methods:**

Concentrations of bictegravir were measured in plasma and breastmilk of healthy, lactating women without HIV after a single dose of bictegravir 50 mg (co-formulated with tenofovir alafenamide 25 mg and emtricitabine 200 mg). Concentrations were measured using validated LC-MS/MS assays and pharmacokinetic parameters were calculated using non-compartmental analysis. Breastmilk to maternal plasma ratio, daily infant dosage and relative infant dose were established to determine infant exposure to bictegravir through breastmilk.

**Results:**

Twelve volunteers participated in the study. The geometric mean (CV%) area under the curve based on the last measured concentration was 52.17 (22.6) mg*h/L in plasma and 0.44 (32.0) mg*h/L in breastmilk, resulting in a geometric mean (CV%) breastmilk to maternal plasma ratio of 0.009 (26.6). The median (IQR) daily infant and relative infant doses with an intake of 150 or 200 mL/kg/day were 0.034 mg/kg/day (0.026–0.060) and 0.046 (0.035–0.080) mg/kg/day and 0.68 (0.53–1.00) % and 0.90% (0.71–1.33), respectively.

**Conclusion:**

Exposure to bictegravir through breastmilk is very low, with a relative infant dose below 1%. Even though metabolizing capacity in newborns is not yet fully developed, it is not expected to cause infant toxicity.

## Introduction

Breastfeeding is widely recognized for its significant health benefits for both mothers and infants and is therefore strongly promoted by the World Health Organization.^1,2^ In the context of maternal HIV infection, the avoidance of breastfeeding has historically been recommended to eliminate the risk of viral transmission through breast milk in settings where safe alternatives, such as formula feeding, are available. However, the risk of HIV transmission is shown to be extremely low when viral replication is effectively suppressed with antiretroviral therapy. As a consequence, current international guidelines^3,4^ support breastfeeding by women living with HIV, provided they maintain a suppressed viral load and demonstrate good adherence to antiretroviral medication.

Despite regulatory requirements for drug labelling to include information on use during pregnancy and lactation, clinically relevant data on newly approved antiretroviral agents often remain unavailable for several years following market authorization.^5^ Recently, data on the use of bictegravir during pregnancy have become available,^6,7^ resulting in a label update by the FDA^8^ and the inclusion of bictegravir as an alternative regimen in pregnancy in international guidelines.^3,4^ Bictegravir has several characteristics that make it particularly suitable for use during pregnancy and the post-partum period, including its formulation as a single-tablet regimen (co-formulated with tenofovir alafenamide 25 mg and emtricitabine 200 mg), once-daily dosing and small pill size. Nevertheless, the extent to which bictegravir is excreted into breast milk remains unknown.

Several studies have investigated the concentrations of various antiretroviral drugs in breastmilk.^9,10^ However, for bictegravir, only limited data exist, with published concentrations derived from three samples obtained from a single individual. Significant intra-individual variability (43%) in these concentrations impedes extrapolation to a broader population and highlights the need for further research to determine whether exposure to bictegravir via breast milk may pose a toxicity risk to infants. The study by Aebi-Popp *et al.* adopted a pragmatic approach by sampling drugs currently used in clinical practice.^10^ Such an opportunistic design is not expected to acquire data within an adequate timeframe for newer drugs such as bictegravir (BIC) 50 mg (co-formulated with tenofovir alafenamide (TAF) 25 mg and emtricitabine (FTC) 200 mg (BIC/FTC/TAF)). It has only recently been approved for use during pregnancy. Also, breastfeeding among women living with HIV have only recently been supported in guidelines. And finally, as lactation data on bictegravir are lacking, physicians are less likely to prescribe it during this period, resulting in low prevalence of bictegravir use among women living with HIV who are breastfeeding. Including healthy, lactating women without HIV infection in clinical pharmacology studies may address this limitation by facilitating more rapid data collection. Therefore, the present study aims to determine bictegravir concentrations in the breast milk of healthy, lactating women without HIV infection following administration of a single dose of BIC/FTC/TAF.

## Materials and methods

This is a pharmacokinetic study in which plasma and breastmilk of healthy, lactating women without HIV were examined after a single dose of BIC/FTC/TAF was administered.

The study was preregistered (NCT05648201) and conducted in accordance with Declaration of Helsinki (2013), Declaration of Taipei (2016), and Dutch Medical Research Involving Human Participants Act (WMO). The trial was approved by the medical ethical committee of East Netherlands (NL83180.091.22). Written informed consent was obtained before study interventions took place.

Healthy, lactating volunteers without HIV who were willing and able to withhold breastfeeding for up to 4 days post-study, to ensure avoidance of unwanted exposure of their infants to contaminated breastmilk, were recruited. As bictegravir is assumed to passively transfer into breastmilk after oral ingestion, the plasma half-life is the main driver for breastmilk half-life. Therefore, participants were instructed to abstain from breastfeeding up to five times the plasma half-life of bictegravir (17 hours), which corresponds with 4 days. So, women who intended to stop breastfeeding or were able to provide alternative feeding (formula, previously expressed breastmilk etc.) while maintaining milk production (using a pump and discarding potentially contaminated milk) were considered eligible for participation. During the prescreening phone call and screening visits, participants were asked about their breastfeeding practices and counselled on how they would feed their infants during study participation. They were encouraged to practice bottle feeding before participation and acquire sufficient alternatives (e.g. formula, pumped milk stored in freezer from before study participation). Also, the option to pump and discard contaminated breastmilk in case of engorgement after intake of study medication and before they are allowed to give breastmilk to their infants. Participants were recruited through adverts in regional and national newspapers and affiliated websites, promotional material in local infant day care facilities and lactation rooms of regional hospitals, and through social media. Exclusion criteria were severe galactose intolerance, HIV infection or co-morbidity or use of co-medication known to interfere with bictegravir pharmacokinetics.

Bictegravir 50 mg is only available in a single-tablet regimen in which it is co-formulated with 25 mg of TAF and 200 mg FTC. After a pre-dose blood and breastmilk sample, a single oral dose of BIC/FTC/TAF was administered. Thereafter, blood samples were collected at 1, 2, 3, 4, 5, 6, 8, 10, 12 and 24 hours post-ingestion and breastmilk samples were collected at 2, 4, 6, 12 and 24 hours post-dosing. Breastmilk samples were collected using an electric pump on both breasts, which were emptied fully at each predefined time point. Samples were homogenized, volume was measured and aliquots were stored at −40°C until the moment of analysis. Participants attended our research department and slept at home (or were provided an overnight stay in a nearby hotel, in cases of long travel distance) during the sampling days.

Plasma and breastmilk drug concentrations were measured with an in-house developed LC-MS/MS assay. The plasma assay is internally and externally validated,^11,12^ and the breastmilk assay is internally validated^13^ according to EMA guidelines.^14^ For both plasma and breastmilk, the lowest and highest levels of quantification for bictegravir were 0.02 and 20.0 mg/L respectively. Pharmacokinetic parameters were determined using non-compartmental analysis performed with Phoenix WinNonlin^®^. The area under the curve of the time–concentration curves were used to determine exposure to bictegravir through breastmilk using formulas depicted in Table 1. In case the percentage of extrapolation for AUC_inf_ was >20%, parameters calculated using the AUC_last_ were also reported. It was presumed that not all participants were exclusively breastfeeding, leading to mixed varying volumes of expressed breastmilk. To determine exposure to bictegravir in cases where cumulative exposure was the highest—when an infant is exclusively breastfed—assumed intakes of 150 and 200 mL/kg were used to extrapolate for these cases, either by multiplying the daily infant dose with a weight adjusted daily intake, or by multiplying the estimated intake (150 or 200 mL) with the breastmilk to maternal plasma ratio (MPR). A relative infant dose (RID) <10% is considered safe according to guidance from the FDA and findings of Versteegen *et al*.^15^

**Table 1.** Formulas used to calculate parameters associated with exposure to bictegravir through breastmilk

Breastmilk: MPR	= AUC_breastmilk_/AUC_maternal plasma_
Absolute daily infant dosage (mg/day)	= Σ (total drug concentration in each milk collection × expressed milk volume in each milk collection)
Daily infant dosage (mg/kg/day) with estimated milk intake of 150 mL/kg/day	= (AUC_breastmilk_/24) × (150 × weight of infant)
Daily infant dosage (mg/kg/day) with estimated milk intake of 200 mL/kg/day	= (AUC_breastmilk_/24) × (200 × weight of infant)
Estimated infant dose (mg/kg/day)	= MPR × (AUC_plasma_/24) × 150 or 200 mL
RID (%)	= Absolute daily infant dosage (mg/kg/day)/maternal dosage (mg/kg/day) × 100
RID (%) with estimated milk intake of 150 or 200 mL/kg/day	= [daily infant dosage with estimated intake of 150 or 200 mL, respectively/maternal dosage (mg/kg/day)) × 100%

## Results

**Figure 1.**
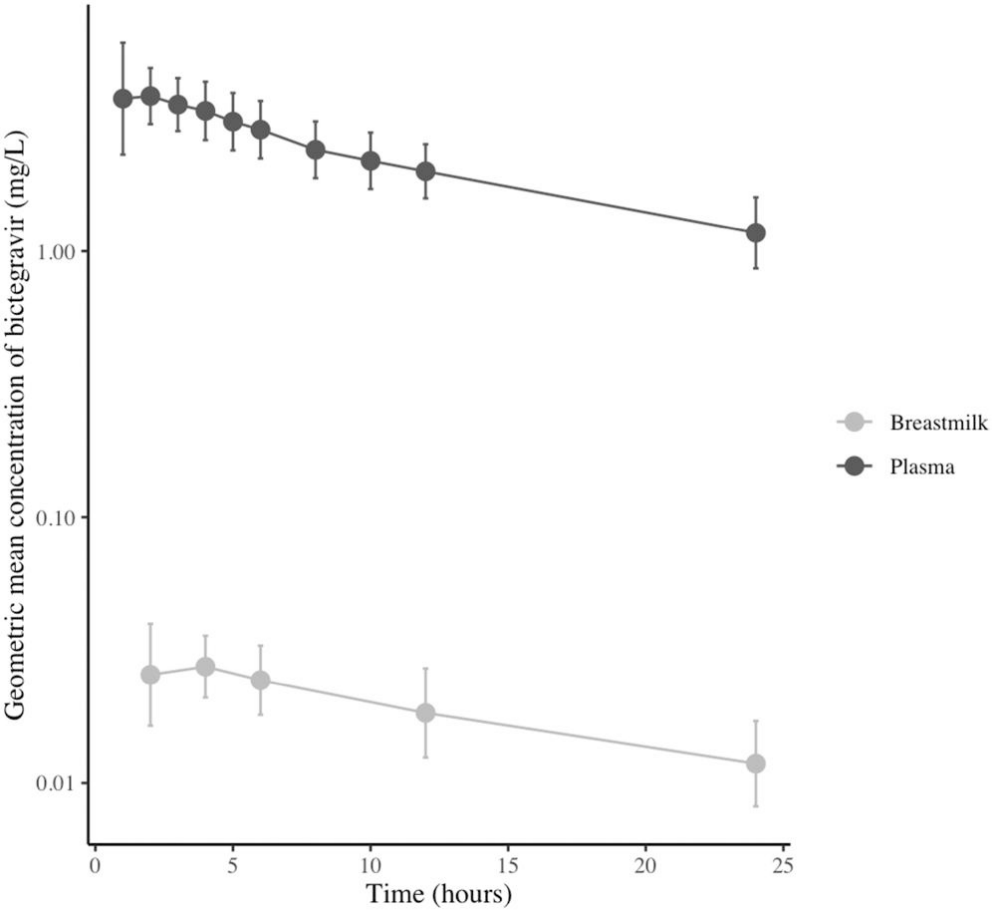
Geometric mean (CV%) concentration of bictegravir (mg/L) in breastmilk and plasma.

Twelve volunteers were included in the study. Their characteristics are listed in Table 2. In total, 132 plasma and 72 breastmilk samples were collected. All concentrations fell within the quantifiable range. The pharmacokinetic parameters in both plasma and breastmilk are listed in Table 3, and the geometric mean (CV%) concentrations of bictegravir in both plasma and breastmilk over time are displayed in Figure 1. Individual time–concentration curves are displayed in Supplemental Figure S1 (available as Supplementary data at *JAC* Online). More than 20% extrapolation was required to determine AUC_inf_ in all participants, therefore, both AUC_inf_ and AUC_last_ are summarized in Table 3. The geometric mean (CV%) AUC_last_ was 52.17 (22.6) mg*h/L in plasma and 0.44 (32.0) mg*h/L in breastmilk, resulting in a geometric mean (CV%) breastmilk to MPR of 0.009 (26.6). The elimination half-life was similar in plasma and breastmilk: that is, a geometric mean (CV%) 15.5 (18.5) hours and 17.2 (28.5) hours, respectively.

**Table 2.** Characteristics of participants

Characteristics	Median (IQR)
Age (years)	33 (32–33.8)
Weight (kg)	78.5 (67.7–82.8)
BMI (kg/m^2^)	24.8 (23.9–28.4)
Time post-partum (months)	10 (5.3–15.3)
Gestational age at delivery	40 (39.8–41)
Weight of infant (kg)	8.8 (7.2–10)

**Table 3.** Bictegravir pharmacokinetic parameters in plasma and breastmilk

Pharmacokinetic parameters	Plasma [GM (CV%)]	Breastmilk [GM (CV%)]
AUC_inf_ (mg*h/L)	79.03 (28.8)	0.75 (40.5)
AUC_last_ (mg*h/L)	52.17 (22.6)	0.44 (32.0)
*C*_max_ (mg/L)	4.634 (17.4)	0.030 (25.8)
*T*_max_ (h)	1.19 (45.2)	3.13 (52.2)
*T*_1/2_ (h)	15.5 (18.5)	17.2 (28.5)

The median (IQR) daily infant dose and RID (%) of bictegravir in this cohort were 0.0077 (0.003–0.012) mg/day and 0.110% (0.05%–0.30%), respectively. When extrapolated to infants that would be exclusively breastfed with 150 or 200 mL/kg/day, the median (IQR) daily infant dosages are 0.034 (0.026–0.060) and 0.046 (0.035–0.080) mg/kg/day, respectively. The RIDs are 0.678% (0.53%–1.00%) and 0.903% (0.71%–1.33%) in infants receiving up to 150 and 200 mL/kg/day. A summary of these exposure parameters is listed in Table 4.

**Table 4.** Bictegravir breastmilk parameters: geometric mean ratio (CV%) or median (IQR)

Breastmilk parameters	GMR (CV%) or median (IQR)
Breastmilk: plasma ratio	
based on AUC_inf_	0.0095 (26.6)
based on AUC_last_	0.0085 (19.4)
Absolute daily infant dose (mg/day)	0.0077 (0.003–0.012)
Estimated infant daily dose (mg/kg/day)	
150 mL/kg/day	0.0378 (0.03–0.06)
200 mL/kg/day	0.0504 (0.04–0.08)
Daily infant dose (mg/kg/day)	
assumed intake of 150 mL/kg/day	0.0344 (0.026–0.060)
assumed intake of 200 mL/kg/day	0.0458 (0.035–0.080)
RID (%)	0.110 (0.05–0.30)
assumed intake of 150 mL/kg/day (%)	0.678 (0.53–1.00)
assumed intake of 200 mL/kg/day (%)	0.903 (0.71–1.33)

No serious adverse events occurred during the study; only grade 1–2 adverse events were recorded (Table S1; Supplementary data), of which one was judged to be probably related to the study medication (mildly elevated bilirubin plasma levels). No concomitant medication was given.

## Discussion

This study demonstrates that bictegravir transfer into breastmilk is minimal, with a breastmilk-to-plasma ratio (MPR) of 0.0085 and a RID <1%. These findings suggest that breastfed infants of mothers treated with bictegravir are unlikely to be exposed to clinically significant drug concentrations. Bictegravir is primarily metabolized by UGT1A and CYP3A4, the activity of which is not fully developed in newborns, possibly leading to bictegravir accumulation and subsequent infant toxicity. However, with the low concentrations measured in breastmilk in this study, ultimate infant exposures are not expected to approach toxic levels. Even though the supposed threshold for a safe RID is arbitrary, severe adverse effects in infants are typically associated with RIDs exceeding 10%,^15^ a threshold far above those observed in this study.

While the risk of toxicity appears negligible, the efficacy of bictegravir in preventing HIV transmission via breastmilk remains uncertain. As this is a single-dose study, steady-state concentrations have not yet been reached. It was not deemed feasible to administer multiple doses and await steady state before sample collection, as this would require participants to abstain from breastfeeding their infants for up to 8 days. Because of this single-dose design, it is not possible to draw firm conclusions about whether therapeutic concentrations are reached in breastmilk. However, concentrations measured 24 hours after intake of bictegravir were below the protein-adjusted IC95 of 1.62 mg/L. In the steady state, the trough levels will probably be higher, but possibly not sufficient for viral suppression. This raises theoretical concerns regarding the potential for resistance development should viral transmission occur.^16,17^ However, in high-income settings, where breastfeeding is only recommended for women with sustained virological suppression and frequent viral load monitoring, the risk of transmission and subsequent resistance is minimal.

The hypothesis that transfer of bictegravir to breastmilk was passive and minimal is supported by data from this study. The similar patterns in time–concentration curves of both plasma and breastmilk suggest that transfer of bictegravir is mainly driven by passive transport. Also, no known drug transporters have been described for bictegravir thus far, nor for other integrase inhibitors. In case of passive transport, high protein binding (>99%) limits passage to the breastmilk compartment. However, diffusion is also facilitated by other factors. Bictegravir is a small molecule (4 714 g/mol), a lipophilic drug (logP 1.28), and a weak acid (p*K*a 8.3), which allows easy transfer and ion trapping. Both fat content and breastmilk pH vary among women, but also within women over the course of time post-partum. These factors underscore the importance of empirical studies to characterize drug transfer, rather than relying solely on physicochemical predictions.

Our findings are consistent with the limited available data on bictegravir in breastmilk, which previously relied on only three samples from a single participant and did not report RID values.^10^ Comparisons with other integrase inhibitors reveal similar trends: dolutegravir exhibits comparable values [median (IQR) MPR 0.04 (0.03–0.05) and median (IQR) RID 1.00% (0.78%–1.33%)], while raltegravir demonstrates greater variability in both parameters [median (IQR) MPR 0.39 (0.39–0.42) for 400 mg BID and 0.96 (0.44–2.68) for 1200 mg QD and respective RIDs 4.09% (2.43%–5.35%) and 0.28% (0.17%–1.42%)].^10^ These comparisons further contextualize the low transfer of bictegravir relative to its pharmacological class.

Several limitations warrant consideration. First, the study used a single-dose design and therefore did not assess steady-state concentrations. However, given bictegravir’s accumulation ratio of 1.54,^18^ it is unlikely that repeated dosing would result in clinically significant increases in infant exposure. Second, as participants were required to abstain from breastfeeding up to 4 days after study participation, this led to the selection of women who were longer post-partum and a large proportion of participants who were no longer exclusively breastfeeding. This resulted in lower milk volumes, potentially leading to underestimation of daily infant dose and subsequent RID. Nevertheless, extrapolation using standardized assumptions for daily milk intake (150–200 mL/kg/day) and incorporating the MPR is an established method for estimating exposure in exclusively breastfed infants.^19–22^ Including exclusively breastfeeding women would have posed significant ethical and practical challenges, making the chosen approach appropriate and consistent with current guidelines.^21^ Last, only exposure to bictegravir was measured, whereas it is unknown what the attribution is of the co-formulated tenofovir alafenamide and emtricitabine. As sufficient data on exposure via breastmilk of emtricitabine^9^ and tenofovir alafenamide^23,24^ are available, it was not measured in this study. Reporting on clinical outcome parameters, alongside exposure, would have been ideal. However, it is not ethical and feasible to expose and subsequently monitor infants of healthy volunteers.^21^ Systematically gathering data in international pharmacovigilance registries (e.g. EudraVigilance from the European Medicines Agency and FAERS from the Food and Drug Administration) would be an alternative option to obtain these data and should therefore be promoted.

The strengths of this study are the innovative approach using healthy volunteers and the use of an intensive pharmacokinetic sampling schedule. Employing healthy, lactating volunteers is only rarely being done, however, as shown in this study, it is a feasible and useful approach to gather lactation data in a timely fashion. In settings where alternative feeding options—such as formula—are not always available, it might be more challenging to apply this approach. However, pre-participation counselling on additional pumping and storing non-contaminated breastmilk before study participation might allow for deployment of this study design in such settings. Another strength is the determination of concentrations for 24 hours enables calculation of pharmacokinetic parameters, especially an accurate MPR. For bictegravir, with its low concentrations, this might not be necessary, however, in other cases where accumulation of drugs does occur in breastmilk, this might allow for developing specific dosing–feeding schedules, minimizing infant risk. We therefore promote this approach, especially for drugs that are known substrates for mammary transporters or are expected to have high breastmilk transfer based on their physiochemical properties.

### Conclusion

Bictegravir does transfer into breastmilk, however, the concentrations in breastmilk are so low that infant toxicity due to exposure to breastmilk of women taking this drug is very unlikely. The use of bictegravir is already approved during pregnancy and this study shows it can also be used in during lactation.

## Supplementary Material

dkag022_Supplementary_Data

## Data Availability

Datasets supporting the results of this study can be made available upon reasonable request.

## Supplementary data

Figure S1 and Table S1 are available as Supplementary data at *JAC* Online.
