# Supplementary material for: Bictegravir concentrations in breastmilk of healthy, lactating women without HIV
Source: J Antimicrob Chemother. 2026 Feb 2;81(3):dkag022. doi: 10.1093/jac/dkag022 (PMC12863268; doi:10.1093/jac/dkag022)
Supplement: dkag022_Supplementary_Data [file dkag022_supplementary_data.docx]

**Supplementary data in separate file**

Table S1 Overview of reported adverse events

| **Participant Id** | **AEs** | **Grade of severity of AE** | **AE related to study drug** | **Action taken** | **Outcome of AE** | **Comments** |
| --- | --- | --- | --- | --- | --- | --- |
| **110009** | Flu like symptoms | 1 | No | No | Recovered | Rhinosinusitis |
| **110018** | Headache | 1 | No | No | Recovered | Headache, which disappeared after a walk outside |
| **110021** | Dizziness | 1 | Unlikely | No | Recovered | Dizziness, which disappeared after heaving a meal |
| **110009** | Headache | 1 | No | No | Recovered | Slept poorly before study participation. Headache resolved after breakfast |
| **110020** | Flu like symptoms | 2 | No | No | Recovered | Rhinosinusitis |
| **110005** | Elevated Bilirubin (35µmol/L – reference range <17 µmol/L) | 1 | Probably | No | Recovered | On day 2 elevated Bilirubin value compared to baseline. One week after study participation bilirubin was 10 µmol/L |
| **110020** | Headache | 2 | Unlikely | No | Recovered |  |


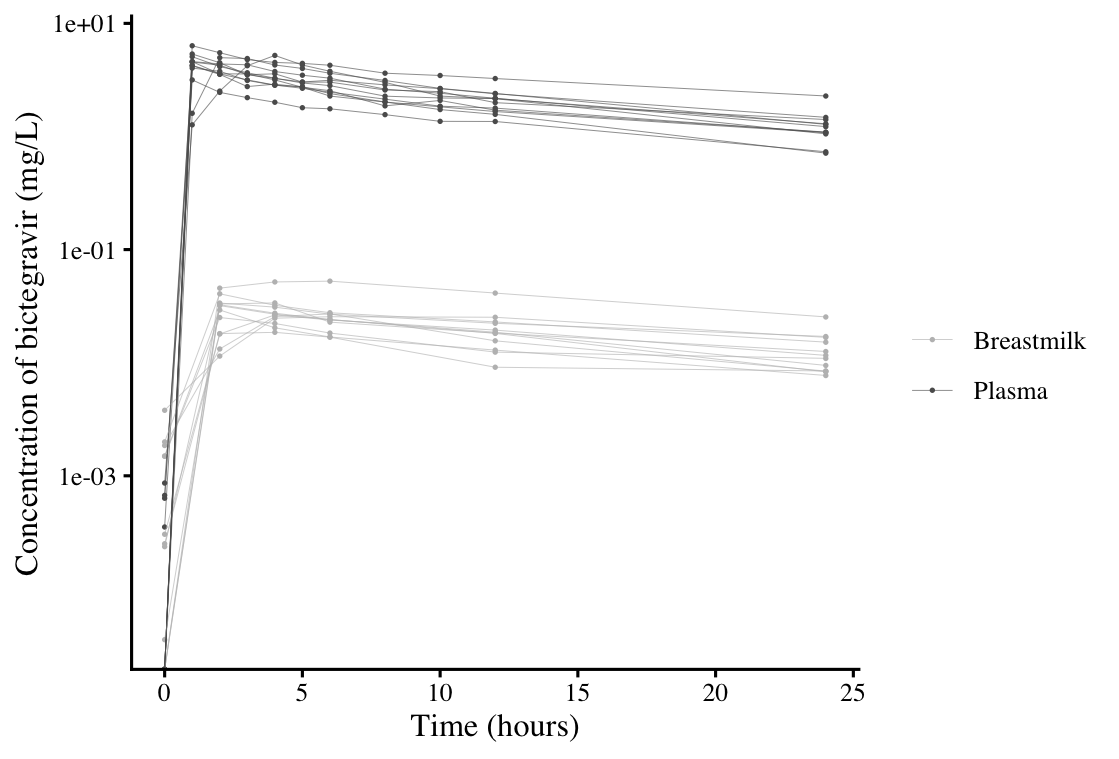


Figure S1 Individual time-concentration curves of bictegravir in plasma and breastmilk
